# Editorial: New mechanistic insight into perinatal origins of reproductive disorders caused by chemical exposures

**DOI:** 10.1016/j.crtox.2022.100089

**Published:** 2022-10-10

**Authors:** Terje Svingen

**Affiliations:** National Food Institute, Technical University of Denmark, Kgs. Lyngby DK-2800, Denmark

**Keywords:** Reproduction, Toxicology, Adverse outcome pathways, Endocrine disrupters

## Abstract

Introducing a Special Issue on mechanism of reproductive disorders in mammals originating from exposure to environmental chemicals during perinatal life.

## Highlights

• Introducing a special research topic on how perinatal exposure to chemicals can cause disease.
• New mechanistic insights into developmental origins of reproductive disorders caused by environmental factors.
• Mechanistic understanding for predictive toxicology, including the Adverse Outcome Pathway framework.

Exposure to chemicals during perinatal life can disrupt reproductive development and ultimately lead to reproductive disorders. This includes disorders manifesting already at birth such as genital malformations, but also late onset disorders such as reproductive cancers and infertility (Skakkebaek et al., 2001). The underlying causes for these diseases can be complex, ranging from gonadal dysgenesis to dysregulated hormone signaling in various target tissues. Since reproductive development is intricately dependent on proper steroid hormone signaling, endocrine disrupting chemicals (EDCs) have received much attention over the past few decades, not least for their potential to disrupt androgen and estrogen signaling.

Broadly speaking, male sexual differentiation depends on a surge in androgen signaling during critical stages of development, whereas female sexual differentiation requires androgen levels to be low during the same developmental stages. This is overly simplified, of course, as estrogens and other hormones also play essential roles in reproductive development of both sexes, as do other signaling pathways and biomolecules. Sexual development relies instead on a complex network of signaling pathways that interact to ensure proper spatiotemporal tissue differentiation and many of these signaling pathways may themselves be vulnerable to disruption by exogenous chemical substances.

To better account for the many ways by which chemicals can disrupt reproductive development, this special issue invited contributions that could shed new light on causal molecular pathways in addition to classical sex hormone signaling. A more long-term vision is that such new insights can be used in a chemical safety assessment and ultimately regulatory decision-making. Especially in a new era of predictive toxicology where chemical risk assessors are asked to rely much more on non-animal test data than on classical animal toxicity studies when determining if a chemical substance may or may not pose a risk to human health.

Disrupted steroid hormone signaling could account for a majority of effect modalities causing reproductive disorders through early-life exposure to chemicals. However, both gonadal differentiation and sexual development rely on additional signaling pathways, for instance the morphoregulatory pathways Wingless-like (WNT), retinoic acid (RA) and Hedgehog (HH). In the first article of this Special Issue, the HH signaling pathway is discussed in the context of reproductive development and disruption by environmental chemicals or drugs (Johansson and Svingen, 2020). Although disrupted HH signaling during embryogenesis can cause severe (or teratogenic) effects, the argument is put forward that more subtle HH disruption at later developmental stages may also contribute to reproductive disorders, not least where HH signaling cross-talk with, for instance, androgen signaling. A particular sensitive tissue in this regard is the genital tubercle, which is the focus of the second article where hypospadias is discussed (Mattiske and Pask, 2021). In their review article, Mattiske and Pask propose that disruption to the androgen-estrogen balance is a main cause of hypospadias arising from exposure to environmental chemicals during development. It is argued that the balance between the two signaling pathways is, in many ways, more important than simply considering an ‘anti-androgenic’ or ‘estrogenic’ mode of action when evaluating causality.

The third article uses the Adverse Outcome Pathway (AOP) framework to construct putative causal toxicological pathways linking developmental exposures to EDCs, particularly phthalates, with malformations of the male reproductive tract (Palermo et al., 2021). The AOP framework is a systematic approach for assembling and evaluating biological knowledge that can aid risk assessors with drawing supporting evidence from mechanistic data, often from alternative test methods, when assessing and managing chemicals that may pose a risk to human or environmental health (Ankley and Edwards, 2018). Albeit putative, the proposed AOP network which includes hypospadias, cryptorchidism and epididymal agenesis that all ultimately converging with impaired fertility, suggest a common mechanistic lineage at the level of steroidogenesis, at least with respect to phthalates. This extensive review of the literature is a valuable source for continued development of encyclopedic AOPs. This literature review also highlights another issue with AOP development, which has been a slow endeavor over the first 10 years from its conception; namely that developing fully endorsable AOP can be extremely time- and resource-demanding. This challenge was recently proposed to be overcome by implementing more pragmatic approaches, including the development, publication and endorsement of smaller units of knowledge such as key event relationships (Svingen et al., 2021).

As a first case for this pragmatic approach to AOP development, the fourth article presents a key event relationship (KER) linking reduced RA signaling in the developing ovary with the failure of oocytes to enter meiosis (Draskau et al., 2022). The RA signaling pathway has long been proposed to be a key target for many environmental chemicals causing endocrine disrupting effects, and as such should receive more focus from a risk assessment perspective (Grignard et al., 2020). The fifth article of this special issue thus rounds off the collection by showing how phthalates can disrupt RA in the mouse testis and cause testicular dysgenesis (Alhasnani et al., 2022). In turn, the consequence of testis dysgenesis can be a failure in hormone synthesis and thus disrupted sexual development, as well as failure in supporting the germ lineage with consequences for adult fertility, or even risk of developing testis cancers.

Since modern toxicology is moving towards relying more on non-animal test strategies for safety assessments of chemical substances, it is imperative that we understand the underlying mechanisms and modes of action that underpin reproductive toxicity (Pistollato et al., 2021). Only then can we confidently use data from alternative test methods or models to assess the risk to human health and ultimately regulate substances based on this knowledge (Svingen et al., 2022). For this to eventuate, much work remains to be done; but hopefully this Special Issue has taken us a little bit further down the right path.

## Disclosure

Given his role as Guest Editor, Terje Svingen had no involvement in the peer-review of this article and has no access to information regarding its peer-review. Full responsibility for the editorial process for this article was delegated to Thomas Knudsen.

## CRediT authorship contribution statement

**Terje Svingen:** Writing – original draft, Writing – review & editing.

## Declaration of Competing Interest

The authors declare that they have no known competing financial interests or personal relationships that could have appeared to influence the work reported in this paper.

## Data Availability

No data was used for the research described in the article.

## References

[b0005] Alhasnani M., Loeb S., Hall S., Caruolo Z., Simmonds F., Spade D. (2022). Interaction between mono-(2-ethylhexyl) phthalate and retinoic acid alters 1 Sertoli cell development during fetal mouse testis cord morphogenesis. Curr. Res. Toxicol..

[b0010] Ankley G.T., Edwards S.W. (2018). The adverse outcome pathway: a multifaceted framework supporting 21st Century toxicology. Curr. Opin. Toxicol..

[b0015] Draskau M.K., Ballegaard A.S.R., Ramhøj L., Bowles J., Svingen T., Spiller C.M. (2022). AOP Key Event Relationship report: Linking decreased retinoic acid levels with disrupted meiosis in developing oocytes. Curr. Res. Toxicol..

[b0020] Grignard E., Håkansson H., Munn S. (2020). Regulatory needs and activities to address the retinoid system in the context of endocrine disruption: The European viewpoint. Reprod. Toxicol..

[b0025] Johansson H.K.L., Svingen T. (2020). Hedgehog signal disruption, gonadal dysgenesis and reproductive disorders: Is there a link to endocrine disrupting chemicals?. Curr. Res. Toxicol..

[b0030] Mattiske D.M., Pask A.J. (2021). Endocrine disrupting chemicals in the pathogenesis of hypospadias; developmental and toxicological perspectives. Curr. Res. Toxicol..

[b0035] Palermo C.M., Foreman J.E., Wikoff D.S., Lea I. (2021). Development of a putative adverse outcome pathway network for male rat reproductive tract abnormalities with specific considerations for the androgen sensitive window of development. Curr. Res. Toxicol..

[b0040] Pistollato F., Madia F., Corvi R., Munn S., Grignard E., Paini A., Worth A., Bal-Price A., Prieto P., Casati S., Berggren E., Bopp S.K., Zuang V. (2021). Current EU regulatory requirements for the assessment of chemicals and cosmetic products: challenges and opportunities for introducing new approach methodologies. Arch. Toxicol..

[b0045] Skakkebaek N.E., Rajpert-De Meyts E., Main K.M. (2001). Testicular dysgenesis syndrome: an increasingly common developmental disorder with environmental aspects. Hum. Reprod..

[b0050] Svingen T., Villeneuve D.L., Knapen D., Panagiotou E.M., Draskau M.K., Damdimopoulou P., O'Brien J.M. (2021). A Pragmatic approach to adverse outcome pathway development and evaluation. Toxicol. Sci..

[b0055] Svingen T., Schwartz C.L., Rosenmai A.K., Ramhøj L., Johansson H.K.L., Hass U., Draskau M.K., Davidsen N., Christiansen S., Ballegaard A.R., Axelstad M. (2022). Using alternative test methods to predict endocrine disruption and reproductive adverse outcomes: do we have enough knowledge?. Environ. Pollut..

